# Linking social attention to reproductive state and future reproduction in female monkeys

**DOI:** 10.1007/s00265-026-03801-w

**Published:** 2026-10-02

**Authors:** Raisa Hernández-Pacheco, Alexis A. Diaz, Nalina Aiempichitkijkarn, Natalia Camargo Peña, Sheida Yahyazadehmoghaddam, Jacob Weber, Elizabeth Maldonado, Shripad Tuljapurkar, Alexandra G. Rosati

**Affiliations:** 1 Department of Biological Sciences, California State University, Long Beach, CA USA; 2 Department of Biology, Stanford University, Stanford, California USA; 3 School of Biology, Suranaree University of Technology, Nakhon Ratchasima, Thailand; 4 Department of Psychology, University of Michigan, Ann Arbor, Michigan USA; 5 Department of Anthropology, Stony Brook University, New York, USA; 6 Caribbean Primate Research Center, University of Puerto Rico-Medical Sciences Campus, San Juan, Puerto Rico USA; 7 Departments of Psychology and Anthropology, University of Michigan, Ann Arbor, MI USA

**Keywords:** Attention bias, Emotional processing, Primate cognition, Social behaviour, Social attention, Reproductive success

## Abstract

The demands of complex social life are thought to drive the evolution of cognition in primates. One important approach to testing this hypothesis is to assess if variation in psychological processes impacts an individual’s fitness outcomes, but this can be challenging in primates where field experiments are rare. Here, we examined a naturalistic, semi-free-ranging population of rhesus macaques (*Macaca mulatta*) where routine cognitive experiments are possible. We tracked cognitive performance and annual reproductive outcomes in this seasonally-breeding species, and tested whether attention to and processing of conspecific social information is (1) affected by reproductive status, and (2) associated with individual variation in reproductive success. Across three annual birth seasons, we experimentally measured attention to unfamiliar conspecific face stimuli in *N* = 103 female monkeys. Reproductive status strongly influenced females’ cognitive performance, as nursing females showed reduced interest in conspecific stimuli compared to those that did not breed. To disentangle current reproductive status effects from relationships between cognition and reproductive outcomes, we then tested whether an individual’s interest in conspecifics was associated with their next-year reproductive success. We found that females that showed greater interest in conspecific faces had a higher likelihood of reproducing in the following year. Our work provides evidence of complex links between sociocognitive performance and reproductive outcomes in monkeys, and highlights how life history stage or physiological state across the life course may also influence the expression of cognitive and behavioural traits.

**Supplementary Information:**

The online version contains supplementary material available at https://doi.org/10.1007/s00265-026-03801-w.

## Significance statement

Appropriate attention to and processing of relevant social information is proposed to impact animals’ biological outcomes. We examined the links between social cognition and reproduction in female rhesus macaques, a highly social and seasonally breeding monkey species. We experimentally measured individual differences in sociocognitive performance and tracked reproductive outcomes over three years. Nursing females (with a young infant) showed less interest in unfamiliar conspecifics relative to females that did not successfully breed that season. In order to account for this relationship between current reproductive state and reproductive outcomes within the same birth season, we then tested associations with future reproductive success and found that interest in unfamiliar females predicted reproduction regardless of current reproductive state. Our results reveal that the complex links between social cognition and reproductive outcomes may be shaped by an individual’s physiological or behavioural state.

**Supplementary Information:**

The online version contains supplementary material available at https://doi.org/10.1007/s00265-026-03801-w.

## Introduction

The social intelligence hypothesis predicts that selection favours cognitive and brain substrates that allow animals to cope with the challenges of social life, such as tracking experiences and relationships with different social partners (Byrne and Bates 2007; Dunbar and Shultz 2007; Silk 2007; Ashton et al. 2018b). However, testing this proposal is challenging. Many studies aiming to evaluate the social intelligence hypothesis have used comparisons of brain size as a proxy for cognition, rather than direct assessments of cognitive skills (Shultz and Dunbar 2007; DeCasien et al. 2017; Powell et al. 2017; Speechley et al. 2024). Other work has directly assessed social cognitive abilities, but typically compared group-level skills across species or populations varying in social structure or complexity in order to evaluate if differences in general social milieu are related to cognitive performance (Herrmann et al. 2007; Reader et al. 2011; Sandel et al. 2011; MacLean et al. 2014; Speechley et al. 2024; Schwob et al. 2025). A key prediction of this hypothesis is that individual variation in cognitive skills is under selection and therefore cognitive performance should be related to fitness outcomes within a population (Morand-Ferron et al. 2016; Ashton et al. 2018b; Madden et al. 2025). Accordingly, some recent work has directly assessed how individual variation in cognitive performance is linked to survival and reproduction in wild populations of birds and mammals (Holt-Lunstad et al. 2010; Cauchard et al. 2013; Steptoe et al. 2013; Morand-Ferron et al. 2016; Ashton et al. 2018a; Huebner et al. 2018; Fichtel 2022; Welklin et al. 2024). However, much of this work focused on non-social aspects of cognition, such as learning, spatial memory, and inhibitory control, rather than social cognitive processes per se. There is therefore a gap concerning how social cognition in particular influences fitness outcomes in animals.

For selection to shape cognition, individuals should exhibit variability in cognitive skills, and this variation must relate to fitness outcomes (Thornton and Lukas 2012; Rowe and Healy 2014; Thornton et al. 2014; Seyfarth and Cheney 2015). Addressing individual-level characteristics to evaluate how social cognition shapes fitness patterns is crucial for understanding primate behavioural evolution, given that primates have complex social systems compared to many other taxa (Smuts et al. 2019; Clutton-Brock 2021). Primates also exhibit long-term, differentiated social relationships and extended periods of parental care that are characteristic of humans, making them an important model for human cognitive and behavioural evolution (Silk 2007; Snyder-Mackler et al. 2020; Shultz and Dunbar 2022). Yet studying the fitness consequences of cognition in primates is challenging as most cognitive testing in primates occurs in captive populations that are managed by humans and do not experience free-breeding conditions where reproductive success can be meaningfully measured. Conversely, studies of cognition in wild primates are limited due to ethical and practical concerns (Zuberbühler 2014; Rosati et al. 2022; Sabbi et al. 2026).

In this study, we investigated whether attention to and processing of conspecific faces is linked to reproductive outcomes in macaques. We focused on face processing as our target sociocognitive trait because recognizing and responding to face stimuli is a foundational process for many different aspects of social cognition and behaviour in both humans and other animals (Adolphs 2003; Leopold and Rhodes 2010; Hugenberg and Wilson 2013; Freiwald et al. 2016; Crivelli and Fridlund 2018). For example, primate social behaviour depends on detecting conspecific faces and facial expressions (Ghazanfar and Santos 2004; Parr et al. 2005; Hoffman et al. 2007; Tsao et al. 2008; Parr 2011) and such facial expressions are a key form of social communication (Parr et al. 2005; Waller et al. 2024). Indeed, facial expressions are proposed to play a crucial role in the formation of primate social bonds (Dobson 2012; Whitehouse et al. 2024), and convey important information about an individual’s potential future actions (Leopold and Rhodes 2010; Waller et al. 2016). As such, accurate detection of other’s facial expressions is a skill that would plausibly provide several evolutionary benefits. We specifically examined these links between face processing and reproductive outcomes by leveraging the unique strengths of the Cayo Santiago Biological Field Station, a large population of semi-free-ranging rhesus monkeys (*Macaca mulatta*) that live in naturally formed, freely-breeding social groups with an extensive record of patterns of reproduction (Hernández-Pacheco et al. 2016). Yet unlike wild primate populations, these animals are also well-habituated to human observers such that they can be routinely tested on controlled experiments to assess individual cognitive performance (Mandalaywala et al. 2014; Drayton and Santos 2016; Rosati et al. 2016, 2018; Diaz et al. 2025).

We used this system to test the hypothesis that attention to conspecific face information is associated with greater reproductive success in female rhesus monkeys. Rhesus macaques are a highly social species, and prior work has shown that patterns of sociality influence key biological outcomes in this population. For example, individuals with larger social networks or more tolerant social relationships show greater survivorship (Brent et al. 2017; Testard et al. 2024; Pavez-Fox et al. 2025). Furthermore, females can face an exacerbated risk of aggression and injury in both the mating and birth season, suggesting that social interactions may affect important reproductive outcomes (Pavez-Fox et al. 2025). Rhesus monkeys also have rich repertoires of facial expressions that are used in social interactions (Parr et al. 2005; Hoffman et al. 2007; Parr and Heintz 2009). Threat expressions and displays in particular are an important signal that monkeys produce in aggressive behavioural contexts, and prior work shows that monkeys discriminate threat from other expressions and that threat expressions are particularly salient (Maestripieri and Wallen 1997; Partan 2002; Gothard et al. 2007; Hoffman et al. 2007; Parr and Heintz 2009; Parr et al. 2010; Bethell et al. 2012). As such, we predicted that monkeys that showed greater interest in conspecific faces (e.g., looked at them for longer periods) would gain reproductive benefits by being more likely to successfully reproduce in a given year. We further assessed the degree to which individuals showed differential looking to conspecific threat faces compared to neutral faces, to test if individual variation in responsivity to threat signals predicted reproduction.

Assessing these links between cognition and reproduction in female primates is complicated by a related question: whether a females’ reproductive state impacts their cognitive performance. Recent work has highlighted that reproductive state effects may account for some previously reported relationships between female animals’ social behaviour and reproductive outcomes. Specifically, a recent re-analysis showed that female baboons with a live infant were more social than females without an infant (Creighton et al. 2025), demonstrating that both presence of an infant and the mother’s reproductive state drove reported relationships between female sociality and infant survival (Silk et al. 2003). In fact, when accounting for this confound, there was no clear relationship between maternal social behaviour and infant survival (Creighton et al. 2025). Accordingly, it is increasingly recognized that linking maternal behaviour to reproductive outcomes requires careful consideration of how reproductive state can have complex effects on behavioural traits (e.g., Feder et al. 2025; Feldblum et al. 2025). Similarly, there is accumulating evidence that matrescence (e.g., transition to motherhood) may affect cognitive performance in humans (Orchard et al. 2023), and some parallel evidence from nonhuman primates concerning how hormones like estrogens or reproductive parity may impact cognitive performance and related behavioural tendencies (Maestripieri and Wallen 1995; Lacreuse and Herndon 2003; Lacreuse et al. 2007, 2015; Waitt et al. 2007). Yet the impacts of reproductive state on nonhuman cognition is an understudied phenomenon because many primate populations where cognitive testing is routine also have human-managed reproduction, as mentioned previously. This indicates that it is important to address potential reproductive states effects in any work linking cognition to maternal reproductive outcomes in a freely-breeding population.

As rhesus macaques are seasonal breeders (unlike baboons, chimpanzees, and many other well-studied primate species), a key aspect of our study is that female monkeys were often pregnant or nursing at the time of their experimental test because our sampling period necessarily overlapped with their birth season. Moreover, monkeys that were not pregnant or nursing were generally nonbreeders for that entire year, given this annual breeding system. In the current work, we therefore first test if rhesus monkeys show reproductive state effects on cognitive performance, and then assess relationships between cognition and future reproduction while excluding such effects. To do this, we implemented a longitudinal field experiment using a preferential looking task that captures individual differences in attention to conspecific images, paralleling prior work on socioemotional processing in humans using naturalistic viewing patterns (Isaacowitz et al. 2006a; b). Preferential looking studies are a key methodology that has been used to assess pre-verbal infants’ developing attention to and processing of face stimuli (Slater et al. 1998; Mondloch et al. 1999; Libertus et al. 2017), and similar methods have also been used in non-verbal primates (Higham et al. 2011; Dubuc et al. 2016; Simpson et al. 2019). Here, we used a previously validated task (Rosati et al. 2018; Diaz et al. 2025) where paired conspecific photographs (the same monkey producing a neutral followed by a threat expression) were presented in a sequence, and measured duration of looking to each photograph, similar to other looking time studies in this population measuring looking to a sequence of events (Drayton and Santos 2016). To address reproductive state effects in this species, we first examined whether cognitive performance as measured in our experiment varied across a female’s reproductive state (e.g., gestating, nursing, or nonbreeding). After directly testing for such reproductive state effects, we then assessed whether performance was linked to reproductive outcomes accounting for the possible effects of reproductive state on current cognitive performance. To do so, we tested if current cognitive performance impacted future (next-year) reproduction, avoiding the association between current reproductive state and same-year reproduction.

## Methods

### Preregistration

We preregistered our experimental cognitive data collection procedures (see https://aspredicted.org/jj9b6.pdf and https://aspredicted.org/n9cw-xwz4.pdf; note that additional aspects of this preregistered plan are not the focus of this study, this paper rather uses cognitive performance to predict reproductive outcomes).

### Study population

All data for this study were collected between 2021 and 2023 as part of the experimental data collection or general longitudinal monitoring of the free-ranging rhesus macaques at the Cayo Santiago Biological Field Station of the University of Puerto Rico. The Cayo Santiago population consists of over 1700 rhesus monkeys living in multiple naturally formed social groups on a 38-acre island off the coast of Puerto Rico (Kessler and Rawlins 2016). Monkeys have ad libitum access to water and commercial monkey chow (0.23 kg/animal/day). Animals are habituated to human observers and a variety of cognitive experiments (Flombaum and Santos 2005; Drayton and Santos 2016; Rosati et al. 2016, 2018) and are individually identifiable based on a combination of unique tattoos (three-character codes), ear notches, and aspects of physical appearance. This species exhibits a promiscuous mating system with annual seasonal breeding, and on Cayo Santiago the population showed reproductive synchrony with over 82% of births occurring within a 4-month period in the summer every year of our study. We conducted cognitive tests within an approximately two‐month testing window during each summer period, attempting to locate the same set of target adults living in two of the groups on Cayo Santiago that were the focus of this longitudinal testing (groups F and V). While we also collected data from males as part of the larger study, here we focus on data from females to evaluate the relationship between cognition and reproductive outcomes because mothers provide parental care in this species and female parentage is directly observable.

### Participants

A total of 103 individual female macaques of reproductive age (i.e., at least 4 years up to 24 years) participated in at least one cognitive test in our longitudinal field experiment characterizing their responses to conspecific socioemotional stimuli. In the final sample, 25 females participated in one year of testing, 38 in two years of testing, and 40 in all three years of testing. As described in more detail below, the experimental test involved up to four trials presenting two pairs of matched photos. In each year, our criterion for inclusion was that the individual stayed in the testing area and attended to at least the first two trials (presenting matched female images). Thus, all 103 participants successfully completed the first two trials for a total of 221 tests involving the female photographic stimuli. Of these monkeys, 82 of them also completed the subsequent pair of trials (presenting matched male images) in at least one year of testing, for a total of 146 tests involving the male photographic stimuli (for details see Supplementary Table S1).

### Overview of field experiment and trials

**Fig. 1:**
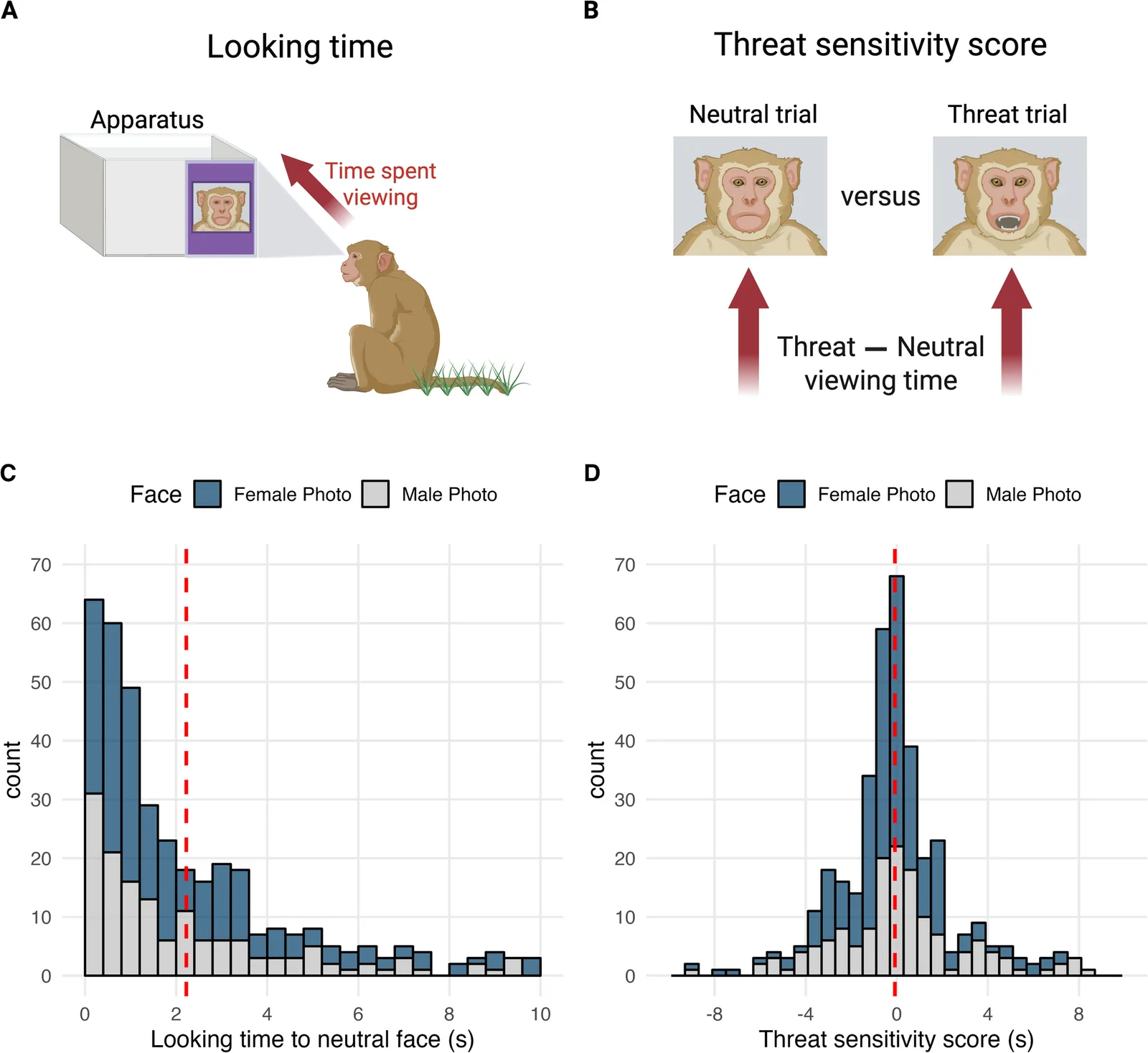
Social cognitive task and individual variation in performance. (**A-B**) Schematic overview of the validated social cognitive task. Preferences for viewing photographs of conspecifics producing emotional versus neutral expressions were measured. Monkeys were presented with up to four photos: a neutral female face (trial 1), followed by a threat face of the same female (trial 2), and a neutral male face (trial 3), followed by a threat face of the same male (trial 4). Each trial lasted 10s. We measured relative social interest as the time spent viewing the neutral expression, and attention bias to the threat versus neutral photos by calculating a threat sensitivity difference score (Threat – Neutral Viewing time). (**C**) Distribution of looking times to neutral female (dark colour) and male (light colour) conspecific faces across trials. (**D**) Distribution of threat sensitivity difference scores for female (dark colour) and male (light colour) faces across trials. Dashed line indicates the population average across trials

To measure social cognition, we conducted a longitudinal field experiment to characterize individual variation in responses to a sequence of conspecific photographs involving either neutral or socioemotional expressions (Fig. 1A-B; Supplementary Fig. S1). This task involved looking time methods, where individual’s cognitive processes are characterized by measuring their looking to different stimuli (Winters et al. 2015; Drayton and Santos 2016). Here we used a validated task that measures responses to conspecific socioemotional signals (Rosati et al. 2018; Diaz et al. 2025). During a test session, subjects viewed up to four photographs in succession: they first viewed a photo of an unfamiliar conspecific female monkey producing a neutral expression (Trial 1), followed by a photo of the same female producing a threat expression (Trial 2). If the subject stayed in the testing area for subsequent trials (e.g., they did not choose to leave or were not disrupted by other monkeys in this free-ranging context), then they viewed a second set of photos: one showing an unfamiliar conspecific male monkey producing a neutral expression (Trial 3) followed by a photo of the same male producing a threat expression (Trial 4).

We included both female and male photographs in the sequence because rhesus monkeys are a female-philopatric species (males are the dispersing sex, whereas females stay with kin in their natal group) and further show major sex biases in social behaviour such as aggression (Bernstein and Ehardt 1986; Bercovitch 1997; Kulik et al. 2015a; b; Pavez-Fox et al. 2025). As such, individuals might plausibly respond differently to threats from females versus males. Indeed, prior work with this task indicated that rhesus monkeys show different patterns of looking to female versus male images, with relatively increased interest in male threat (Rosati et al. 2018; Diaz et al. 2025). As we were primarily interested in individual variation in responses, we always presented the stimuli in a fixed order in sessions. While counterbalancing of stimuli presentation is typically used when work is aimed at detecting differences between conditions or stimuli, studies in experimental psychology that are focused on individual differences have different methodological considerations, and counterbalancing produces spurious individual differences related to presentation order (see Cronbach 1957; Mollon et al. 2017; Hedge et al. 2018; Goodhew and Edwards 2019). In this case, looking time tasks specifically often produce habituation-related decline in looking over successive trials, introducing potentially large variation related to order assignment rather than true individual variation in psychological traits. In order to better capture true individual variation in cognitive processes without introducing additional variation in responses related to stimuli order, we therefore implemented a fixed order of stimuli presentation. This followed recommendations and approaches commonly used in experimental psychology studies with humans across cognitive domains (Hedge et al. 2018; Andermane et al. 2019; Luzardo and Yeshurun 2021; White et al. 2022; Growns et al. 2024; Garner and Robison 2025), as well as prior work with monkeys in this population focused on individual differences (Rosati et al. 2018; Huang et al. 2023; Diaz et al. 2025; Schwob et al. 2025).

In the task, we measured monkeys’ looking time to each photo, and then extracted metrics of (1) overall interest in conspecific faces and (2) attentional biases for relative looking towards matched threat versus neutral expressions, (see Fig. 1A-B for a schematic overview and Supplementary Fig. S1 and Supplementary Videos S1 and S2 for experimental demonstration). Specifically, this threat sensitivity difference score characterized each individual’s relative looking to the threat versus matched neutral face, thereby capturing individual differences in responsivity to threat while accounting for within-individual variation in overall interest in faces. Such difference scores are commonly used in looking time studies with both infants and animals (Connellan et al. 2000; Oakes 2017; Goupil et al. 2024) to capture preferential looking patterns. In this population, prior work found that while monkeys show declines in looking to female threat photographs compared to the matched neutral stimuli, they show increased looking to male threat, as well as overall increases in the threat score with age (Rosati et al. 2018; Diaz et al. 2025)—indicating modulation of responses to conspecific threat may depend both on which is the signaller and which is the receiver.

We aimed to test subjects on this task at three time-points, each one year apart, to link cognitive performance to annual reproductive outcomes and further characterize within-individual variation in social interest over time. Thus, females could participate in the task for up to three years if they survived and were successfully located for testing in each testing period. In the free-ranging context of Cayo Santiago, monkeys may not have been located in an appropriate context to present the stimuli in a given year (e.g., sitting calmly in a location where we could present the apparatus to them), or could have been excluded because they were displaced during testing and did not complete the study (see details on exclusions below). Because the order of trials was fixed, subjects were only included in analyses of responses to male photographs if they completed all four trials. Thus, our analysis characterizing responses to male faces was restricted to the subset of subjects tested on both stimuli pairs.

### Experimental procedure and apparatus

In test sessions, two experimenters walked around the island searching for target monkeys that were sitting calmly and located in an appropriate position where the experimenters could present the apparatus with the socioemotional stimuli. For example, monkeys in inappropriate positions included those that were sitting high in a tree, on a cliff, or where their eyes were not clearly visible due to light conditions because we could not present the apparatus so they could see it, or we could not film their looking patterns to be able to code it. The primary experimenter positioned themself approximately 2–3 m away from the monkey, kneeling on the ground with an apparatus (34 cm high, 16 cm deep, 54 cm wide) that allowed them to present the series of photographs through a front-facing window (23 cm wide, 30 cm tall; Supplementary Fig. S1). The second experimenter knelt behind them to film the monkey’s face so we could measure their looking time.

At the start of each trial the window was covered by a flap. The primary experimenter first attracted the monkey’s attention to the box by calling the monkey (e.g., “monkey, monkey!”) and/or tapping the apparatus. When the primary experimenter judged the monkey was looking to the apparatus during the live experiment, they initiated the trial by lifting the flap so that the monkey could see the photograph, and also simultaneously saying “now” (this cue was used during video coding to mark the start of the trial). Whether the monkey was looking at the start of the trial was also confirmed by blind coders in the subsequent coding phase. When the experimenter presented the photograph, they also adverted their gaze downwards to avoid eye contact with the monkey during the ten second trial. After ten seconds elapsed, the camera person said “stop” to mark the end of the trial. Between trials, the primary experimenter replaced the flap and exchanged the photos, and then quickly started the next trial in the sequence by again attracting the monkey’s attention back to the apparatus. As the study involved within-subject comparisons to the matched neutral and threat trials for the female and male photograph pairs, monkeys had to successfully stay in their location, attending to and observing the stimuli for both trials in a pair to be included in the relevant analyses, as noted above.

As with prior implementation of this task, we assessed preferences for viewing photographs of unfamiliar conspecifics (i.e., monkeys from other groups from the Cayo Santiago population rather than individuals from that monkey’s current group or birth group that they plausibly know). To accomplish this, there were two possible photo sets presented to the monkeys as stimuli, each comprising matched photos of a given adult female and adult male producing a neutral and threat facial expression; the age of the female and male individuals approximately matched across the two sets. While we generally limited the number of exemplars since we were primarily interested in capturing individual variation in responses, we had two photosets in order to present most subjects with a set of unfamiliar conspecifics where the subject did not share the photo monkey’s natal or current group. Since tested monkeys had origins in multiple groups, it was not possible to present unfamiliar stimuli using only one set. We then assigned each monkey to a given photoset based on their birth and current group so that they observed photographs from unfamiliar individuals. While three females did see photographs from monkeys they potentially knew, additional analyses reported below indicated that they did not differ in their responses, as there was no effect of the presentation of outgroup versus ingroup photographs on responses, as in prior work using this task (Rosati et al. 2018; Diaz et al. 2025).

### Exclusions

In this free-ranging context, monkeys were necessarily tested when other monkeys were also present in the vicinity. As such, some monkeys were approached for testing but failed to produce a scoreable response, typically because they did not complete all necessary trials to be included. Usually, this happened because the monkey (1) chose to walk away from the testing area before completing the first two trials; (2) they were displaced by other monkeys before doing so. For example, social interactions between other monkeys in the group may have disrupted the monkey’s participation in this free-ranging context, such that the monkey did not complete the minimum required trials to be included (i.e., at least the first two trials involving female stimuli). In addition, some sessions were excluded because (3) the video was not possible to accurately score (e.g., due to video backlighting, or because the monkey was judged to be not looking at the apparatus at the start or the trial, or moved out of the camera shot so their face was not visible to score). If possible, these monkeys were approached for testing again on a later date. In addition to the 221 test sessions included in the final sample, we performed but excluded an additional 37 sessions based on these criteria (*N* = 8 sessions were excluded in year 1, *N* = 6 in year 2, and *N* = 23 in year 3); these exclusion rates were similar to or less than exclusion rates in comparable prior studies in this population (Rosati et al. 2018; Diaz et al. 2025). In some cases, target animals were approached more than once for testing because we only confirmed their ID after the test session was complete. In this situation, we only included and analysed their first successful session in that year in the final data set.

### Video coding and reliability

We followed standard procedures for coding monkey looking time data (e.g., Huang et al. 2023; Diaz et al. 2025; Schwob et al. 2025). Specifically, two experienced, independent coders who were blind to trial type coded the cognitive data from each year to assess whether the monkey was looking at the apparatus over the 10s trial. To do so, individual trials were clipped from longer video sessions, and randomized with a random numeric code label such that coders were blind to trial type, and different trials from the same monkey were interspersed across the full set of clips with no identifying cues. A given trial clip started 1–2 s before the experimenter one said “now” to allow coders to better judge where the monkey was looking (i.e., eye position) when the experimenter started the trial as the reference. We coded each trial for 10s from the starting point (see Supplementary Video S1 for example coding clips). Coders examined clips frame-by‐frame using the program Filmora to assess the monkeys’ looking responses, based on the monkey’s head and eye position (visualizable given the contrast between rhesus monkeys’ paler iris and pupils); note that in this context where they could freely move about, monkeys typically looked at the apparatus by orienting both their head and eyes towards the apparatus, and similarly often looked away by overtly changing their head orientation or even entire body. As these data were collected over several years there were different coders depending on the year, and there was high reliability across the two coders each year (Year 1 Pearson’s *r* = 0.93; Year 2 Pearson’s *r* = 0.98; Year 3 Pearson’s *r* = 0 0.93).

### Demographic data

We extracted demographic data on the monkeys that completed the cognitive tasks using the Cayo Santiago’s database. These data were collected via systematic visual censuses by Cayo Santiago staff that take place five days a week and record the date of birth, maternal ID, sex, group membership, and date of death or permanent removal from the population of all individuals immediately or usually within two days after the event occurred (Ruiz-Lambides et al. 2017). Our primary analyses focused on reproductive outcomes defined by successful live births since most infants in this population survive to the juvenile period (Gonzalez et al. 2023). We specifically extracted data on whether female subjects produced a live offspring during the annual birth season a year prior to our test (i.e., prior reproduction), during the season concurrent with our test (i.e., same-year reproduction), and during the season a year after our test (i.e., next-year reproduction). Information of same- and next-year reproduction were conditional on female survival. All females surviving past the 99-percentile date of the annual distribution of births were included in the analysis (Supplementary Fig. S2). This allowed us to maximize sample size by disregarding isolated birth events at the end of each season. We note that while population density is an important factor driving reproductive outcomes in female monkeys at Cayo Santiago (Bland et al. 2025), density during the study period was similar and thus unlikely to be an important driver of variation in reproduction.

This information also allowed us to retrospectively assign subjects one of three reproductive states at test date: nursing (i.e., caring for a live newborn at test), gestating (i.e., pregnant at test), and nonbreeding (i.e., not gestating and not nursing at test). Within the context of our study, our nonbreeding reproductive state classification is independent of whether a female was reproductively active or not (e.g., in principle some nonbreeders may not have mated that year), but given that all female subjects were of reproductive age, and the vast majority had had at least one pregnancy prior to this study (except 4 individuals), this is unlikely in such an annually-breeding species. As such, all individuals could potentially reproduce one time in each year of the study. Viable pregnancies in rhesus macaques can range from 147 to 180 days (Coe and Lubach 2021), and the longest time between a test and an offspring birth in our data was 130 days (Supplementary Fig. S3). Thus, we were able to use the test date and the offspring birth date to determine which subjects were gestating at test date based on later observed birth events. Although there is a possibility to misclassify some gestating females as nonbreeders (e.g., those experiencing the end of an unobserved pregnancy), historical records of the annual proportion of stillbirths and abortions are low (< 5%; Rawlins and Kessler 1983). In our sample, only two subjects were observed having stillbirths, but these events happened within a 1–2 month period after the test date and thus we assumed both females were gestating at test date. Females that produced a live birth before the experimental test, but which infant did not survive to the test date (i.e., failed breeders) were assigned the reproductive state of “nonbreeding”. Notably, only five subjects in seven occasions were failed breeders and excluding them from our analysis did not generally change conclusions (see Supplementary Table S18-19). These females were therefore included in our main analysis.

### Statistical analysis

We performed all analyses in R version 4.4.2 (R Core Team 2024) and implemented generalized linear mixed-effects models (GLMMs) using package glmmTMB (Brooks et al. 2017). To account for repeated measurements, all models included individual identity as random intercepts. We also conducted post hoc Tukey comparisons of hypothesized model factors and estimated marginal means (i.e., population means adjusted across factors and covariates) using package emmeans (Lenth 2024). We checked for correlation among predictors (Supplementary Table S2, S3) and multicollinearity (variance inflation factor $\:\le\:$ 2.50; see Supplementary Information: Model diagnostics) using package performance (Lüdecke et al. 2021), and model assumptions on homogeneity and normality of residuals using package DHARMa (Hartig 2022; see Supplementary Information: Model diagnostics). All numerical predictors were Z-standardized.

We first examined overall patterns of responses in the experimental task. Given the different patterns of looking to the female versus male stimuli reported previously (Rosati et al. 2018; Diaz et al. 2025), as well as the fact that all female subjects completed the first two trials showing female photos but not necessarily the last two trials showing the male photos, we implemented separate analyses of responses to the female stimuli versus the male stimuli across the entire study. Following prior work with this task (Rosati et al. 2018; Diaz et al. 2025), we assessed both *total looking time* to conspecific photographs across the sequence of stimuli, as well as the *threat sensitivity difference score* (Threat looking time–Neutral looking time). In this score, positive values indicate relative increased interest in or attention to the threat face, negative scores indicate relative declines, and scores near 0 indicate similar attention regardless of valence. This score is therefore independent from overall interest in faces, which is important because individuals tend to show correlations in looking across successively presented trials (e.g., a monkey that looks for a long duration at the first photograph likely also shows long total looking to the next one) (Rosati et al. 2018; Diaz et al. 2025). This allows us to compare the importance of overall looking and threat bias as distinct predictors in subsequent analyses.

To characterize looking time to conspecific photographs in these models, we fitted GLMMs with a log-link gamma distribution as recommended for right-skewed data (Lo and Andrews 2015). The models included the additive linear effect of *age* and the factors *facial expression* (neutral vs. threat), *outgroup* (i.e., whether the set of photographs the monkey saw belonged to a conspecific living outside their group), and *photoset* (i.e., which specific set of photographs the monkey saw in the test) as predictors. Variables accounting for outgroup and photoset were included as factors in models because they were experimentally manipulated variables, and prior work with this paradigm in this population indicated that both can impact looking time responses in the population (Rosati et al. 2018; Diaz et al. 2025). While stimuli exemplars (e.g., the photoset variable in the current study) are sometimes treated as random effects in psychological studies (Judd et al. 2012), this was not possible here given that photoset was a factor with only two levels; we discuss the implications of this modelling approach in later sections. Following prior work that identified relevant interaction effects using a subset of our data (2021–2022; Diaz et al. 2025), we also tested whether including interaction effects between photoset and facial expression in the male stimuli model improved model fit using likelihood ratio test. Although such relationship is not part of our hypotheses, we were interested in accounting for any relevant effect of the experimental design on the dependent variable. As a significant improvement in model fit was observed, the interaction term was retained and supplementary model summaries using effect-coded variables reported. To characterize the threat sensitivity difference score, we implemented GLMMs with a Gaussian error distribution and included the additive linear effect of *age* and the factors *outgroup* and *photoset* as predictors.

We next evaluated the relationship between reproductive state and social cognition by testing if reproductive state predicted task performance, while also accounting for age effects and relevant experimental variables. To contrast between current reproductive state effects, which may involve nursing an infant, and the presence of an immature but weaned offspring, we also tested for associations with the presence of a live yearling. First, we tested associations between overall looking time and reproductive state by fitting GLMMs with a log-link gamma distribution. The models included the additive linear effect of *age*, and the factors *facial expression*, *reproductive state*, and *presence of a live yearling* as predictors. In the male stimuli model, we also included the main effect of *photoset* and the interaction between *photoset* and *facial expression* as predictors given its statistical significance found when examining overall patterns of socioemotional attention. We then similarly tested associations between the threat sensitivity difference score and reproductive state. For this, we fitted GLMMs with Gaussian error distribution. The models included the additive linear effect of *age*, *reproductive state*, and *presence of a live yearling* as predictors. In the male stimuli model, we also included *photoset* as predictor to test if the specific photo exemplars that the monkeys experienced impacted their responses.

Following these initial checks, we then evaluated the hypothesized relationship between socioemotional attention and reproduction. For this, we tested whether the probability of reproduction is linked to cognitive performance, while also accounting for age effects and prior reproductive performance. Here, we used looking time to the neutral face as our metric of overall social interest, and the threat sensitivity difference score as a metric of emotional responses. As noted above, the threat score captures sensitivity to threat above and beyond a general interest in face stimuli, so this approach allowed us to test both as conceptually independent predictors of reproduction in the same model. To implement these models, we fitted GLMMs with a binomial error distribution and a logit link function for the binary outcome of whether a female produced a live offspring in the following birth season after the experimental test and added the additive linear and quadratic effects of *age*, the factor *skipped* describing current reproductive state (i.e., whether the female skipped the current birth season), and the two hypothesized cognitive variables (*looking time to the neutral face* and *threat sensitivity score*) as predictors. We included the “skipped” variable to capture current reproductive performance, rather than reproductive state as in the looking time models, because these models assessed later reproductive outcomes as the dependent variable rather than cognitive performance at the same time the monkey was experiencing a specific reproductive state. Accordingly, whether the monkey reproduced or not in the current year is a more relevant predictor of subsequent reproduction, as almost all monkeys that reproduced experienced both pregnancy and nursing states (excepting those that experienced a stillbirth). In addition, the ‘skipped’ variable captures the majority of the relevant variance related to the reproductive status (see Supplementary Table S10).

## Results

### Patterns of socioemotional attention

Longitudinal analyses of cognitive performance showed substantial individual variation in monkeys’ attention to conspecific stimuli and their relative attention biases to threat versus neutral expressions (Fig. 1C-D). Across *N* = 221 test sessions, subjects viewed the neutral female photograph an average of 2.22 ± 0.15s (mean ± SE; range: 0.07 to 10s) and the threat female photograph an average of 2.01 ± 0.16s (range: 0.03 to 9.90; see Supplementary Fig. S4). Across the *N* = 146 test sessions where subjects also completed the male photograph trials, they viewed the neutral male photograph an average of 2.23 ± 0.19s (range: 0.03 to 9.60s) and the threat male photograph an average of 2.34 ± 0.20s (range: 0.03 to 9.90). Subjects showed an average threat sensitivity difference score of -0.21 ± 0.15s (range: -8.70 to 7.27s) when looking at female photographs. This negative average score indicates overall less looking at the female threat stimuli compared to the initial matched female neutral expression. Conversely, subjects showed an average difference score of 0.11 ± 0.24s (range: -8.77 to 8.47s) when looking at male photographs, indicating relative increases in looking at male threat compared to the initial neutral male stimuli. The females in the current study exhibited overall declines in interest in conspecific images with age (e.g., declines in overall durations of looking), and relative increases in sensitivity to male threat stimuli (see details in Supplementary Table S4-S9), which is in line with prior work showing overall declines in looking but also increasing attention to threat specifically (Rosati et al. 2018; Diaz et al. 2025). This shows that we detected relevant variation across individuals in both their general social interest and emotional attentional biases previously reported in studies with larger mixed-sex subject samples.

### Reproductive state and socioemotional attention

**Fig. 2:**
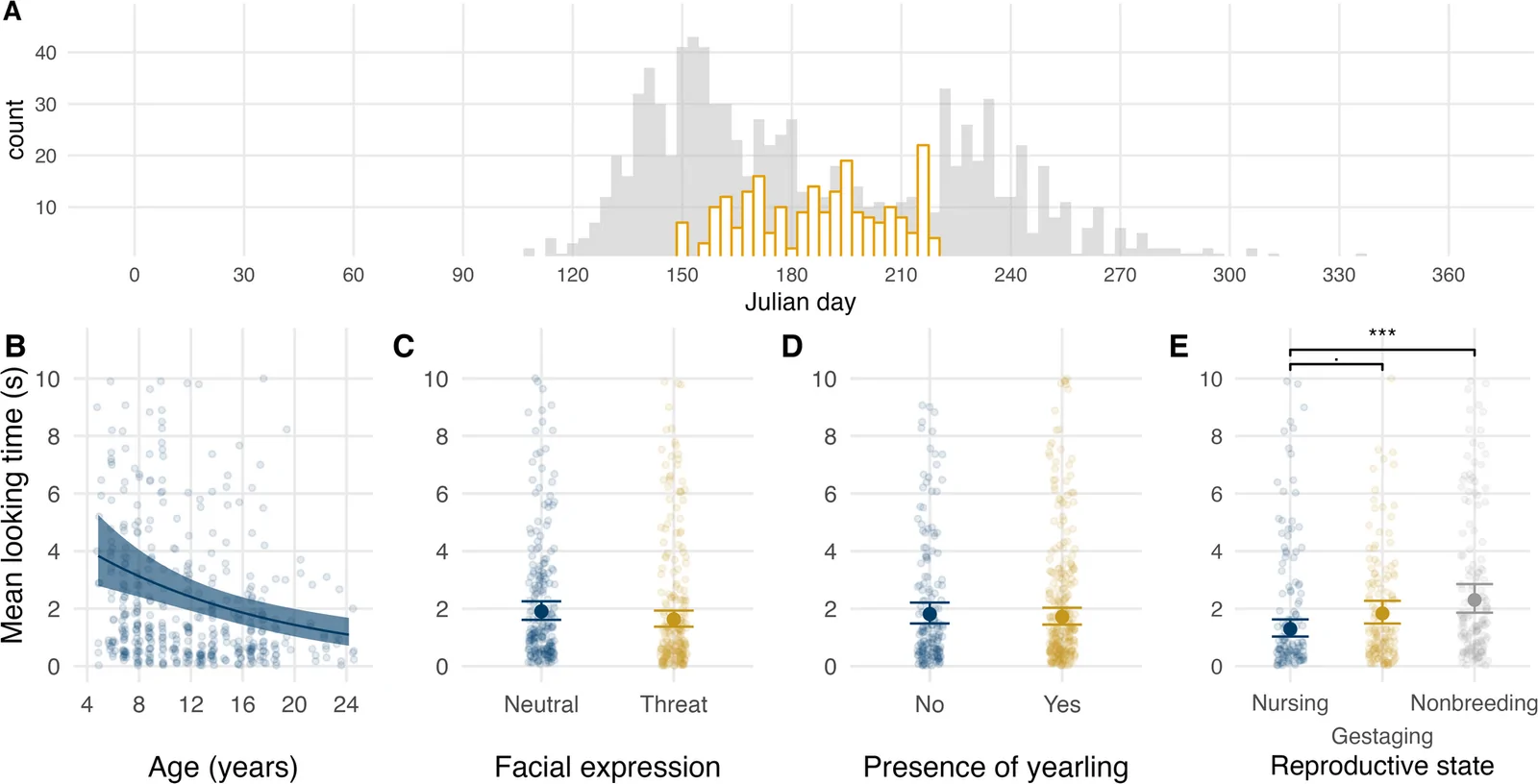
Association between reproductive state and socioemotional attention. (**A**) Date distribution of offspring births (grey) and cognitive tests (white) of female rhesus macaques from 2021 to 2023, and average predictions for mean looking time towards female stimuli as a function of (**B**) age and across (**C**) facial expression, (**D**) presence of yearling, and (**E**) reproductive state. Average predictions of factors in **C**-**E** represent population means adjusted across factors and covariates. “⋅” and “***” indicate marginal significance and significance at the 0.05 and < 0.001 levels, respectively. Ribbons and error bars represent 95% confidence intervals. Data points represent empirical data

Across the 221 test sessions examining responses to the female photos, 66 subjects were nursing females, 77 were gestating females, and 78 were nonbreeding females (Supplementary Table S1). Nursing females looked at the female neutral photos for an average of 2.13 ± 0.29s, while females that were gestating or did not breed looked an average of 1.91 ± 0.21s and 2.61 ± 0.28s, respectively. Nursing females looked at the female threat photos for an average of 1.77 ± 0.29s, while females that were gestating and did not breed looked an average of 1.69 ± 0.22s and 2.53 ± 0.29s, respectively. In fact, our analysis revealed that reproductive state at the time of the experimental test was significantly associated with sociocognitive performance; on average, gestating ($\:\beta\:=0.350;p=0.020)$ and nonbreeding ($\:\beta\:=0.577;p<0.001)$ females showed increased attention to female conspecifics, relative to nursing females (Table 1; Fig. 2). Post-hoc pairwise comparisons also showed a significant difference in looking time between nursing and nonbreeding females ($\:\beta\:=-0.577;p<0.001)$, and a marginal difference in looking time between nursing and gestating females ($\:\beta\:=-0.350;p=0.053$) when averaging effects across all factors (Supplementary Table S11). After adjusting for facial expression, presence of yearling, and age, population means for nursing, gestating, and nonbreeding females looking at the female photos were 1.29 (95% CI: 1.03, 1.62), 1.84 (1.48, 2.27), and 2.30 (1.86, 2.85), respectively (Fig. 2E). We found no evidence suggesting that the presence of a yearling influenced sociocognitive performance ($\:\beta\:=-0.056;p=0.636$; Table 1; Fig. 2), indicating that this shift in attention was specifically related to having a newborn in the postpartum period, not caring for any young dependent offspring.

When examining responses to the male photos, nursing females (*N* = 45 testing sessions) looked at male neutral photos for an average of 2.14 ± 0.38s, while females that were gestating (*N* = 49 testing sessions) and did not breed (*N* = 52 testing sessions) looked an averaged of 2.23 ± 0.32 s and 2.30 ± 0.31s, respectively. Nursing females looked at male threat photos for an average of 2.04 ± 0.29s, while females that were gestating and did not breed looked an average of 2.12 ± 0.31 s and 2.81 ± 0.41s, respectively. Here, our analysis revealed marginal evidence that reproductive state at the time of the experimental test was significantly associated with sociocognitive performance (Table 1; Table S13-S15): nonbreeding females showed increased interest in unfamiliar male conspecifics, relative to nursing females ($\:\beta\:=0.365;p=0.048$). That is, as with the pattern of responses we observed for female stimuli, nonbreeding females showed enhanced attention to unfamiliar conspecifics. However, post-hoc pairwise comparisons of the model showed no significant differences when averaging effects across all factors and covariates (Supplementary Table S12). After adjusting for facial expression, presence of yearling, and the age covariate, population means for nursing, gestating, and nonbreeding females looking at the male photos were 1.56 (95% CI: 1.18, 2.06), 1.83 (1.38, 2.43), and 2.25 (1.74, 2.90), respectively. We also found no evidence suggesting that the presence of a yearling influenced sociocognitive performance ($\:\beta\:=-0.097;p=0.525$; Table 1), as for the female photos.

**Table 1:** Relationship between reproductive state and socioemotional attention. Coefficients of the models describing relationships between looking time to conspecific photographic stimuli and experimental variables, age, reproductive state, and presence of a yearling of female rhesus macaques. Models include the random intercept of individual ID. All numerical predictors are Z-standardized. Brackets represent the factor level, and “X” represents an interaction

Variable	estimate	SE	*p*
*Female stimulus *(*N* = 442 trials)			
intercept	0.363	0.122	< 0.010
facial expression [threat]	-0.157	0.088	0.075
age	-0.306	0.073	< 0.001
reproductive state [gestating]	0.350	0.151	0.020
reproductive state [nonbreeding]	0.577	0.149	< 0.001
presence of yearling [yes]	-0.056	0.117	0.636
*Male stimulus* (*N* = 292 trials)			
intercept	0.398	0.174	0.022
photoset [2]	0.277	0.189	0.144
facial expression [threat]	0.424	0.158	0.007
age	-0.188	0.081	0.020
reproductive state [gestating]	0.157	0.190	0.408
reproductive state [nonbreeding]	0.365	0.185	0.048
presence of yearling [yes]	-0.097	0.152	0.525
photoset [2] X facial expression [threat]	-1.018	0.240	< 0.001

When examining the threat sensitivity score derived from responses to female conspecific images, nursing females showed an average threat sensitivity score of -0.36 ± 0.27s; while females that were gestating and did not breed showed an average score of -0.21 ± 0.28s and − 0.08 ± 0.25s, respectively. Our analysis indicated no associations between reproductive state and threat sensitivity (Supplementary Table S16). For male photos, nursing females showed an average threat sensitivity difference score of -0.10 ± 0.40s, whereas gestating and nonbreeding females looked an average of -0.11 ± 0.37s and 0.51 ± 0.48s, respectively. However, we also found no evidence for associations between reproductive state and the threat sensitivity score and between presence of a yearling and threat sensitivity score (Supplementary Table S16-S17).

### Socioemotional attention and next-year reproduction

On average, 67% of female subjects produced a live offspring each year. Given our result that nursing females tended to show reduced interest in conspecific faces, and nursing females at the time of the test were necessarily mostly successful breeders for the year in this annually breeding species, these reproductive state effects would necessarily drive any association between social attention and reproductive outcomes during the same year. Indeed, 100% of nursing females and 97% of gestating females had a live birth in the concurrent birth season of the cognitive test, while only 10% of females that were categorized as nonbreeders at the time of the test had a live birth in that year. As such, a female’s reproductive state at the time of the cognitive test was directly related to that monkey’s same-year reproductive outcome. To address our hypothesis that attention to conspecific face information is associated with greater reproductive success, while also accounting for these reproductive state effects, we therefore examined if current cognitive performance predicted next-year reproduction. Importantly, there is no relationship between current and next-year reproduction in these monkeys ($\:{\beta\:}=0.417;p=0.413$; Table S20): in this provisioned population with distinct mating and birth seasons, females can typically return to breeding status in the following year regardless of whether they reproduced the prior season. In that way, using the experimental test to predict next-year reproduction addresses reproductive state effects.

**Fig. 3:**
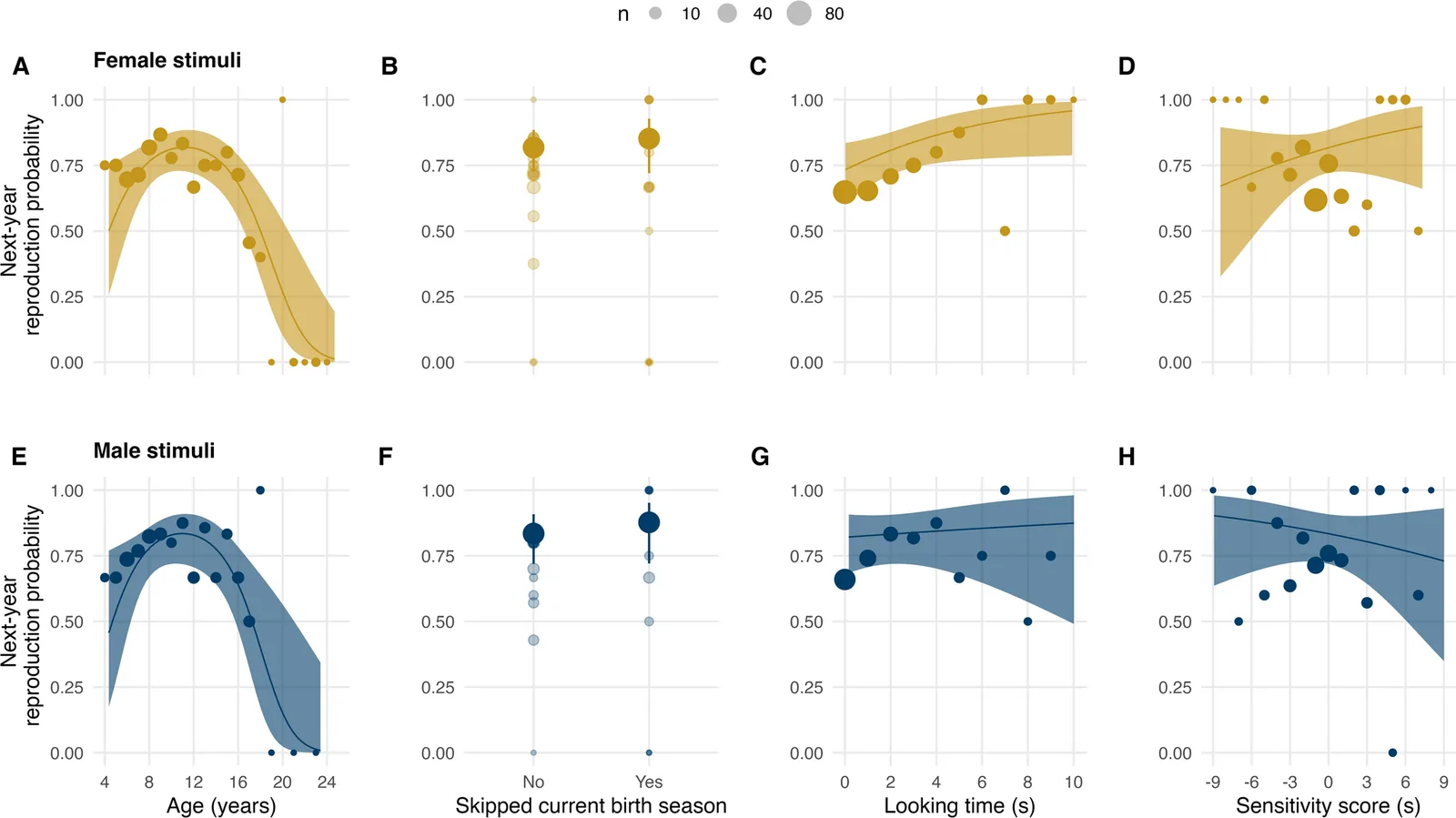
Social cognition is positively associated with next-year reproduction in monkeys. Predictions of the models describing associations between next-year probability of reproduction and demographic and cognitive variables in female rhesus macaques looking at female stimuli (top panels) and male stimuli (bottom panels). Panels **A**-**D** show the effects of age, prior reproductive performance, looking time to a neutral face, and threat sensitivity of the female stimuli model. Panels **E**-**H** show the effects of age, current reproductive performance, looking time to a neutral face, and threat sensitivity of the male stimuli model. Ribbons and error bars represent 95% confidence intervals. Data points represent empirical probabilities, and their size *n* represents sample size per discrete age value (panels **A**-**B**, **E**-**F**), per discrete looking time value (panels **C**, **G**), and per discrete threat sensitivity score value (panels **D**, **H**)

In fact, analyses indicated that duration of looking at the female neutral face was positively associated with the likelihood of next-year reproduction (Table 2). These models accounted for the monkeys’ age as well as their current reproductive state via the *skipped* variable, and found that increased social interest in female conspecifics predicted that monkeys were more likely to produce an offspring during the next birth season ($\:\beta\:=0.477;p=0.045$; Table 2; Fig. 3). We found a significant association with the quadratic term of age ($\:{\beta\:}^{2}=-0.723;p=0.001$) but not with current reproductive performance ($\:\beta\:=0.244;p=0.534$). We found no associations between the female threat sensitivity score and the probability of reproduction the following year ($\:\beta\:=0.213;p=0.296$; Table 2), indicating that this pattern was driven by overall interest in the female photo. Finally, our analysis of (the smaller set of) responses to the male photographs revealed no association between socioemotional attention metrics and next-year reproduction (Table 2). That is, we found that specifically attention to female conspecific faces, as indexed by the monkeys’ looking durations to the initial image they saw, predicted their successful reproduction the following year.

**Table 2:** Likelihood of next-year reproduction based on social cognitive performance. Coefficients of the models describing the probability of next-year reproduction as a function of demographic and cognitive variables. “2” denotes a quadratic term. Models include the random intercept of individual ID. Brackets represent the factor level. All numerical predictors are Z-standardized

Predictor	coefficient	SE	*p*
*Female stimulus* (*N* = 199 test sessions)			
intercept	1.478	0.275	< 0.001
age	-0.226	0.188	0.228
age^2^	-0.723	0.226	0.001
skipped current birth season [yes]	0.244	0.392	0.534
looking time to neutral face	0.477	0.238	0.045
threat sensitivity score	0.213	0.204	0.296
*Male stimulus* (*N* = 136 test sessions)			
intercept	1.614	0.348	< 0.001
age	-0.005	0.224	0.981
age^2^	-0.704	0.274	0.010
skipped current birth season [yes]	0.350	0.477	0.463
looking time to neutral face	0.098	0.270	0.716
threat sensitivity score	-0.206	0.255	0.420

## Discussion

Our longitudinal approach experimentally measured socioemotional attention in female rhesus monkeys over three years and evaluated links between cognitive performance, reproductive state, and reproductive outcomes. Our study revealed strong reproductive state effects on cognitive performance: females that were nursing and thus were taking care of a newborn at the time they completed the experimental test showed less interest in unfamiliar conspecifics than did either pregnant or nonbreeding females. To account for these reproductive state effects when analysing reproduction, we therefore tested whether cognitive performance in a given year is linked to that monkey’s next-year reproductive outcome. Since next-year reproduction is unrelated to current-year reproduction in this population, reproductive status at the time of the test was therefore dissociated from future reproductive success. We found some support for our hypothesis: interest in the initial female conspecific image was positively linked to next-year reproductive success; conversely, there was no relationship between interest in the subsequently presented male stimuli and reproductive success. Overall, these results show that while cognition may be linked to reproductive outcomes, reproductive state also can profoundly impact some aspects of cognitive performance. Tests of the relationship between cognition and reproduction must therefore account for these biologically relevant contextual effects. More generally, our work suggests that performance on experimental sociocognitive tasks can be related to key fitness outcomes in monkeys, aligning with the proposal that individuals that are more motivated and capable of engaging with others will have favourable biological outcomes (Seyfarth and Cheney 2015; Kavanagh et al. 2024; Whitehouse et al. 2024).

While some studies have previously looked at the fitness consequences of cognition in animals, our study has several key differences from prior work. First, we focused on sociocognitive performance metrics that are directly related to sociality, rather than non-social skills like learning and memory that have been the focus of much prior work on cognition and fitness (Pritchard et al. 2016; Ashton et al. 2018a; Pravosudov 2022; Vámos and Shaw 2022; Welklin et al. 2024). Second, while much work testing the fitness benefits of cognition in wild populations has focused on survival (Huebner et al. 2018; Rochais et al. 2022; Welklin et al. 2024), we rather examined reproductive success, as in some prior work with birds (Cauchard et al. 2017; Ashton et al. 2018a; Shaw et al. 2019). This is an important consideration as different facets of cognition may have distinct relationships with different aspects of fitness, such that the cognitive skills that are related to longevity may not be the same skills that are related to successful reproduction. At Cayo Santiago, there is some prior work demonstrating that social relationships predict survivorship (Brent et al. 2017; Testard et al. 2024), so an important question for future work is whether aspects of social cognition are also related to survivorship. Most prior work linking cognition to fitness has examined birds, or else in fairly asocial mammals such as dwarf lemurs (Fichtel 2022), highlighting the importance of also testing social cognitive performance in highly social species with long-term social bonds, such as macaques. Finally, our analysis highlights the importance of considering maternal state effects when examining how maternal traits impact fitness in animals like primates, which exhibit extended periods of investments in pregnancy and post-partum maternal care. We note that while the positive relationship between social attention and future reproductive success was statistically significant, and holds even while accounting for other relevant predictors of reproduction such as age, it also falls just below the significance threshold of *p* < 0.05. Yet we emphasize that this is also one of the first attempts to examine social cognitive associations with reproduction in a freely breeding primate, adding to a growing body of work on social components of fitness more broadly. This approach therefore sets the stage for future work examining the fitness consequences of other important social cognitive traits, such as gaze following (Sabbi et al. 2026) or theory of mind (Crockford et al. 2012; Royka and Santos 2022).

Why are some aspects social attention linked to reproductive outcomes? We examined responses to face stimuli as these cognitive processes are thought to be foundational to many more complex aspects of social cognition and behaviour. One likely possibility is that our cognitive metrics reflect, in part, individual differences in monkeys’ more general tendencies to respond appropriately to social signals (Whitehouse et al. 2024). As competent facial and emotional processing is a necessary precondition for many aspects of social cognition and behaviour (Adolphs 2003; Leopold and Rhodes 2010; Little et al. 2011; Hugenberg and Wilson 2013; Freiwald et al. 2016; Crivelli and Fridlund 2018), animals that show greater social interest and motivation in face stimuli may be more likely to form effective social bonds. Moreover, if macaques can anticipate the actions of others from facial expressions (Waller et al. 2016), it could be beneficial to disengage from partners with threatening expressions in order to evade potential conspecific aggression particularly during mating. While we did not find a clear relationship between the threat sensitivity score and future reproduction in the current study, further work examining how emotional perception impacts different fitness outcomes is warranted. A related consideration for these fitness outcomes concerns whether and to what degree monkeys’ cognitive performance is heritable. Prior work using this looking task shows that monkeys exhibit long-term repeatability in performance, indicating some stable individual differences in cognitive responding (Diaz et al. 2025). This is a key consideration for studies of selection (Cauchoix et al. 2018; Wilson 2018), as repeatability in performance sets the upper bound for heritability. On the other hand, we showed that these cognitive responses are sensitive to reproductive state and thus also can change within-individuals. Thus, a crucial next step to understand the processes of selection on cognition will be to further assess the stability and heritability of these responses.

Notably, our results showed dissociated patterns for how responses to female versus male stimuli relate to reproduction: overall interest in female photographs, but not the male photographs, was related to next-year reproduction. We cannot rule out that the lack of statistical associations for the male stimuli could stem from the reduced sample size for that analysis, as only a subset of the total females participated in the male stimuli trials. We also note that given our fixed trial order—which we implemented to optimize measures of individual variation—all monkeys saw the female photographs first in the sequence; it is possible that these results may have differed had the male photographs had been presented first. Indeed, the lower total looking durations to male photographs likely reflect a habituation process occurring over the sequence of stimuli (although the relative increase in looking at male threat stimuli last in the sequence suggests that the monkeys specifically dishabituated to perceived threats from males). More generally, we note that we only used two sets of stimuli (also to optimize measures of variation) and analysed photoset as a fixed effect, so it is unclear how the current results would generalize to other specific stimuli exemplars. Indeed, our analysis showed that while responses to the two sets of female images were similar, responses to male threat image depended on which specific photoset monkeys saw; this experimental variance may have also impacted the lack of relationships we found for responses to the male stimuli. Conversely, there may be a true distinction between monkeys’ social attention to female versus male partners. Female macaques show stronger bonds with other females, which tend to be their kin in this female philopatric species, and also face a heightened risk of aggression from males (Kapsalis and Berman 1996; Kulik et al. 2015b; Brent et al. 2017). This points to the key role of female relationships and male aggression in shaping an individual monkey’s social landscape. In that way, an important question for future work is whether social attention to same- versus opposite-sex partners actually has different impacts on reproduction.

Our finding that a female’s current reproductive status affects their response to social cues provides a demonstration of such a reproductive state effect on primate cognition in a large, naturally-breeding population. Prior work examining the impact of menstrual cycle stage or oestrogens more generally on cognition in female primates (Maestripieri and Wallen 1995; Lacreuse and Herndon 2003; Lacreuse et al. 2007, 2015; Waitt et al. 2007) has generally focused on smaller samples of animals in laboratory conditions. Here, we were able to compare females in different states (pregnant, nursing, or nonbreeding) by testing cognition in a freely-breeding population where animals’ reproductive behaviour is not manipulated by humans. In fact, nonbreeding females exhibited enhanced attention to unfamiliar adult monkeys especially when compared to nursing females (with similar patterns for images of both sexes). This finding aligns with growing evidence from humans concerning how the transition to motherhood can influence a variety of cognitive and brain functions (Orchard et al. 2023; van ’t Hof et al. 2026). One possibility is that reproductively active female monkeys may face a trade-off between attention to and investment in their own young offspring, versus attention to other adults. In fact, prior work with humans shows that mothers exhibit enhanced attention to infant stimuli compared to adult stimuli, including for facial images like those used here, exhibiting a pattern of selective attention that is different from that observed in non-mothers (Thompson-Booth et al. 2014a;b; Bjertrup et al. 2021). One way to evaluate this possibility in future work would be to present monkeys with contrasting images of conspecific adults versus infants, to explicitly test if breeding mothers actually show enhanced attention to infants as opposed to subdued attention to any conspecific social stimuli. That is, breeding females may have shown decreases in attention to the adult images in the current study due to a selective focus of attention towards offspring.

What are the specific mechanistic processes by which reproductive state affects cognition? That is, given that mothers show shifts in their attentional processes compared to nonbreeders, what drives this shift? One possibility is that the relationships between cognitive performance and reproductive state could stem from hormonal or other physiological states that post-partum females experience. For example, variation in oestrogen levels related to menstrual cycle can impact social preference in monkeys (Lacreuse 2007), so a similar process may be at work here. An important target for future studies would be how cognitive performance relates to shifts in reproductive hormones during pregnancy and in the post-partum period. Alternatively, reproductive state effects could also arise from a shift in behavioural goals: nursing females may be motivated to invest more in social bonds with their offspring than in social interactions with unfamiliar adults. Importantly, we found no influence of the presence of a live yearling on cognitive responses, so the observed decreased in social interest in adult stimuli among nursing females is unlikely to be due to a general effect of caring for any immature offspring. Rather, this shift seems to be specific to the psychological and/or behavioural impact of a newborn or very young infant. In any case, our findings here converge with recent studies from the wild indicating that reproductive state and the presence of infants can shape maternal social behaviour and must be accounted for to understand how selection acts on complex traits (Creighton et al. 2025).

A limitation in our analysis is that we did not include information about individual’s social rank, as we did not collect observations of social interactions necessary to impute these females’ ranks. While social rank can impact fitness in animals in a variety of ways, prior studies examining female monkeys at Cayo Santiago specifically have found mixed evidence that higher dominance rank provides reproductive advantages. While some work in Cayo Santiago showed that rank confers advantages to females through earlier sexual maturation and higher offspring survival rate (Bercovitch and Berard 1993; Blomquist et al. 2011), more recent studies found no evidence that maternal rank influenced the survival of infants (Lee et al. 2020) and no evidence that rank influenced ageing patterns of fertility or reproductive trajectories (Lee et al. 2021). Given that the vast majority of female subjects produced at least one offspring prior to our study, and we accounted for prior reproductive performance in analyses, any rank-related reproductive advantages are unlikely to have had a major effect here. Indeed, since macaques are seasonal breeders where offspring are typically weaned by the end of their first year of life, females may mate and conceive again in the following season with little physiological constraint (Maestripieri and Hoffman 2012). We also note that while prior studies evaluating associations between maternal sociality and fitness has examined effects of sociality above and beyond that effect of rank (Silk et al. 2003, 2009; Kalbitzer et al. 2017; Schneider-Crease et al. 2022; Blersch et al. 2023), it is also the case that primate social behaviour is strongly shaped by rank. However, there is little work to date on whether cognitive performance in primates is similarly related to rank. At Cayo Santiago, one relevant finding is that the infants of both high-ranking and especially protective females show a great bias towards conspecific threat stimuli (Mandalaywala et al. 2014), but it is unclear if the impact of early-life experiences persists into sustained biases in the adult monkeys we tested here. As such, understanding how rank may impact social cognition overall, and whether such rank effects might mediate reproductive outcomes, is an important question for future work.

A more general question for future work concerns the causal pathway(s) linking cognition, behavioural patterns, and biological outcomes. We have argued that our work highlights the importance of cognitive traits as potential mechanisms mediating the link between social behaviour and reproduction that have been more broadly studied in wild animal populations. Yet the relationships between cognition, behaviour, and components of fitness are likely more complex than this. On the one hand, internal cognitive processes do not produce a detectable phenotype except by shifting an organisms’ behavioural patterns so most analyses detecting associations between cognition and fitness assume that the expression of fitness-relevant behaviours are driven by one or more underlying cognitive traits (see Madden et al. 2025 for a review). For example, individual variation in the spatial memory of bird species such as chickadees, as measured in experimental tasks, is thought to impact their caching behaviours and ultimately fitness (Pravosudov and Roth 2013; Vámos and Shaw 2022; Welklin et al. 2024; Pravosudov et al. 2025). Similarly, in the current work it may be that female monkeys with greater social interest (as measured by the looking time task) are more likely to seek out social interactions or have more social bonds, and therefore successfully reproduce in a given season. This kind of causal pathway is supported by evidence that a given cognitive response is repeatable, reflecting a more stable or intrinsic trait of the individual (Branch et al. 2022; Diaz et al. 2025; Semenov et al. 2024). However, behaviour and cognition likely do not show one-to-one or even unidirectional relationships. For example, an individual’s experiences plausibly also impact their cognitive performance, such that behaviour can also drive cognitive responding. For example, females that have more relationships (for any variety of reasons) may show greater looking times to conspecifics due to their different social landscape—which would produce a detectable relationship between cognition and fitness outcomes, but not because of a direct causal relationship. While this reversed pathway may be true of any cognitive skill (for example birds that engage in more caching due to environmental circumstances may show better memory performance due to practice), distinguishing between these interpretations will require not only measuring cognitive traits and fitness (see Madden et al. 2025), but also concurrently assessing behavioural patterns in the same population. While methodologically challenging, such large-scale datasets are increasingly feasible with recent innovations in testing cognition in wild animals (Thornton et al. 2014; Pritchard et al. 2016; Rosati et al. 2022).

In summary, we found that social cognitive performance was strongly shaped by reproductive status and also predicted future reproductive success in a freely-breeding monkey species. Primates have been a major focus of work on social components of fitness recently, including for understanding how aspects of social relationships and behaviour relate to fitness outcomes (Silk 2007; Ellis et al. 2019; Snyder-Mackler et al. 2020; Duboscq et al. 2023; Tung et al. 2023). Several studies examining the links between maternal sociality and reproductive success in primates have tested whether mothers with stronger or numerous social bonds are more likely to have offspring that survive past infancy, or that have longer lifespans, than mothers that have weaker or fewer social bonds (Silk et al. 2003, 2009; Kalbitzer et al. 2017; Schneider-Crease et al. 2022; Blersch et al. 2023; but see Creighton et al. 2025). Here, we tested how relevant psychological processes—interest in conspecifics and attention to conspecific emotional signals—impact successful breeding outcomes in rhesus monkeys. We found relevant variability in social cognitive competencies among individuals, and that greater social interest was linked to a higher likelihood of future reproduction. Future work on how social cognitive performance relates to a given animals’ behavioural strategies, as well as how cognition may be related to survival and other components of fitness, will therefore be crucial to test how cognition and behaviour evolves.

## Supplementary Information

Below is the link to the electronic supplementary material.

Supplementary Material 1

Supplementary Material 2

Supplementary Material 3

## Data Availability

Data and associated R codes are available at Dryad: https://doi.org/10.5061/dryad.qnk98sg0h
