# Supplementary material for: Linking social attention to reproductive state and future reproduction in female monkeys
Source: Behav Ecol Sociobiol. 2026 Oct 2;80(11):131. doi: 10.1007/s00265-026-03801-w (PMC13633143; doi:10.1007/s00265-026-03801-w)
Supplement: Supplementary file 1 [file 265_2026_3801_MOESM3_ESM.docx]

**Supplementary Information for**

**Linking social attention to reproductive state and future reproduction in female monkeys**

Raisa Hernández-Pacheco^1^, Alexis A Diaz^1,2^, Nalina Aiempichitkijkarn^1^, Natalia Camargo Peña^3,4^, Sheida Yahyazadehmoghaddam^1^, Jacob Weber^1^, Elizabeth Maldonado^5^, Shripad Tuljapurkar^2^, Alexandra G Rosati^3,6^

^1^Department of Biological Sciences, California State University, Long Beach, California, United States

^2^Department of Biology, Stanford University, Stanford, California, United States

^3^School of Biology, Suranaree University of Technology, Nakhon Ratchasima, Thailand

^4^Department of Psychology, University of Michigan, Ann Arbor, Michigan, United States

^5^Department of Anthropology, Stony Brook University, New York, United States

^6^Caribbean Primate Research Center, University of Puerto Rico-Medical Sciences Campus, San Juan, Puerto Rico, United States

^7^Department of Anthropology, University of Michigan, Ann Arbor, Michigan, United States

**Corresponding authors:**

**Raisa Hernández-Pacheco**

rai.hernandezpacheco@csulb.edu

Department of Biological Sciences, California State University, Long Beach, California, USA

ORCID: 0000-0002-3681-5127

**Alexandra G Rosati**

rosati@umich.edu

Departments of Psychology and Anthropology, University of Michigan, Ann Arbor, Michigan, USA

ORCID: 0000-0002-6906-7807

**Supplementary Information**

A. **Supplemental methods**
B. **Patterns of socioemotional attention**
C. **Reproductive state and socioemotional attention**
D. **Socioemotional attention and next-year reproductive fitness**
E. **Model diagnostics**

**A. Supplemental methods**

*Sample size*

| **Table S1**. Female rhesus macaque subjects per year. Numbers inside parentheses indicate the subset of female subjects that participated in trials 3 and 4 (completed all trials). | | | | | |
| --- | --- | --- | --- | --- | --- |
| **Year** | **Participants** | **Nonbreeding** | **Gestating** | **Nursing** | **Presence of yearling** |
| 2021 | 83 (56 all trials) | 31 (22) | 32 (20) | 20 (14) | 54 (33) |
| 2022 | 77 (49) | 25 (15) | 18 (12) | 34 (22) | 49 (32) |
| 2023 | 61 (41) | 22 (15) | 27 (17) | 12 (9) | 34 (21) |
| All | 221 (146) | 78 (52) | 77 (49) | 66 (45) | 137 (86) |

*Cognitive task experimental design*

**Figure S1. Setup and stimuli for cognitive task.** (a). Monkeys viewed a series of photographs of conspecific monkeys producing matched neutral or threat expressions. After the monkey attended to the apparatus, the experimenter would remove a posterboard flap to reveal the photograph for that trial, which was displayed for 10s. (b) In the first two trials, monkeys observed matched photos of a female conspecific producing first a neutral expression (left photo) and then the same individual producing a threat expression (right photo). (c) If monkeys remained in the testing area, then they completed a second set of trials with matched photos of a male conspecific producing first a neutral expression (left photo) and then the same individual producing a threat expression (right photo).

*Supplementary videos*

*Video S1: Socioemotional responses task—experimental demonstrations.* This video shows the example experimental demonstration of the procedure. In the first clip, the primary experimenter (the demonstrator) attracts the monkey’s attention to the apparatus and then uncovers the window to reveal a photograph of a female conspecific with a neutral expression. During the timed trials, she holds a position where she looks down with her hat covering her eyes. The second clip shows the same procedure, here uncovering the matched threat photograph from the same individual. In between trials, the experimenter moves the photographs in the apparatus to set up the next trial.

*Video S2: Socioemotional responses task—example monkey responses.* These clips show examples of female macaque looking responses in the task. As illustrated in these videos, clips start a few seconds before the primary experimenter said “now” and did not contain any information about the trial type, such that coders could assess them blind to experimental variables. On the videos, the secondary experimenter (the camera person) then says “stop” after at least 10s have passed; clips were always coded for exactly 10s from the moment the trial started to equate total trial duration across monkeys.

*Distribution of births in Cayo Santiago*

Figure S2. Distribution of births in Cayo Santiago during the 2021, 2022, and 2023 annual birth seasons. The dashed line marks the 99-percentile of birth dates.

Figure S3. Distribution of days between a gestating female subject’s test date and her offspring’s birth date (*N* = 77 gestating females).

*Correlation analysis among predictors:*

| **Table S2**. Pearson correlation among demographic and cognitive metrics for responses to female photographic stimuli (trials 1 and 2, completed by all monkeys); these variables are used as numerical predictors of the probability of reproduction in female rhesus macaques (*N* = 221 test sessions). Bolded correlations correspond to those between model predictors. | | | |
| --- | --- | --- | --- |
|  | **age** | **looking time to neutral face** | **looking time to threat face** |
| **age** |  |  |  |
| **looking time to neutral face** | **-0.284** |  |  |
| **looking time to threat face** | -0.160 | 0.497 |  |
| **threat sensitivity score** | **0.117** | **-0.478** | 0.524 |

| **Table S3**. Pearson correlation among demographic and cognitive metrics for responses to male photographic stimuli (trials 3 and 4, completed by monkeys who remained in the testing area); these variables are used as numerical predictors of the probability of reproduction in the subset of female rhesus macaques looking at a male face (*N* = 146 test sessions). Bolded correlations correspond to those between model predictors. | | | |
| --- | --- | --- | --- |
|  | **age** | **looking time to neutral face** | **looking time to threat face** |
| **age** |  |  |  |
| **looking time to neutral face** | **-0.188** |  |  |
| **looking time to threat face** | -0.137 | 0.230 |  |
| **threat sensitivity score** | **0.035** | -**0.596** | 0.645 |

**B. Patterns of socioemotional attention**

**Figure S4**. Mean socioemotional responses of female rhesus macaques indexed by the duration of looking towards neutral and threat female and male stimuli, broken down by female subjects’ age cohort (Adult 4 to 15 years of age; Old adult: 15-24 years of age). Error bars represent SE.

*Model describing patterns in overall looking time*

| **Table S4.** Coefficients of the models describing patterns in looking time to conspecific photographic stimuli of female rhesus macaques. Brackets indicate the factor level. All numerical predictors are Z-standardized. Bolded *p* values represent statistical significance at the 0.05 level. | | | | |
| --- | --- | --- | --- | --- |
| **Predictor** | **Coefficient** | **SE** | ***z* value** | ***p*-value** |
| *Female stimuli (N = 442 trials)* |  |  |  |  |
| intercept | 1.294 | 0.465 | 2.784 | **0.005** |
| photoset [2] | -0.117 | 0.115 | -1.018 | 0.309 |
| outgroup [yes] | -0.585 | 0.448 | -1.304 | 0.192 |
| facial expression [threat] | -0.139 | 0.090 | -1.543 | 0.123 |
| age | -0.257 | 0.068 | -3.779 | **<0.001** |
|  |  |  |  |  |
| *Male stimuli (n = 292 trials)* |  |  |  |  |
| intercept | 1.288 | 0.589 | 2.187 | **0.029** |
| photoset [2] | 0.294 | 0.188 | 1.567 | 0.117 |
| outgroup [yes] | -0.781 | 0.572 | -1.367 | 0.171 |
| facial expression [treat] | 0.444 | 0.158 | 2.811 | **0.005** |
| age | -0.182 | 0.079 | -2.312 | **0.021** |
| photoset [2] X facial expression [threat] | -1.041 | 0.241 | -4.331 | **<0.001** |

*Effect-coded factors model:*

| **Table S5.** Coefficients of the model describing patterns in looking time to conspecific male photographic stimuli of female rhesus macaques. Brackets indicate the group level. All numerical predictors are Z-standardized, and all categorical variables are effect-coded. Bolded *p* values represent statistical significance at the 0.05 level. | | | | |
| --- | --- | --- | --- | --- |
| **Predictor** | **Coefficient** | **SE** | ***z* value** | ***p*-value** |
| *Male stimuli (n = 292 trials)* |  |  |  |  |
| intercept | 1.006 | 0.289 | 3.477 | **<0.001** |
| photoset [1] | 0.113 | 0.073 | 1.562 | 0.118 |
| outgroup [no] | 0.391 | 0.286 | 1.367 | 0.172 |
| facial expression [neutral] | 0.038 | 0.060 | 0.639 | 0.523 |
| age | -0.182 | 0.079 | -2.312 | **0.021** |
| photo set [1] X facial expression [neutral] | -0.260 | 0.060 | -4.331 | **<0.001** |

| **Table S6.** Coefficients of the main effect only model describing patterns in looking time to conspecific male photographic stimuli of female rhesus macaques. Brackets indicate the group level. All numerical predictors are Z-standardized, and all categorical variables are effect-coded. Bolded *p* values represent statistical significance at the 0.05 level. | | | | |
| --- | --- | --- | --- | --- |
| **Predictor** | **Coefficient** | **SE** | ***z* value** | ***p*-value** |
| *Male stimuli (n = 292 trials)* |  |  |  |  |
| intercept | 1.514 | 0.605 | 2.504 | **0.012** |
| photoset [2] | -0.207 | 0.152 | -1.362 | 0.173 |
| outgroup [yes] | -0.766 | 0.587 | -1.304 | 0.192 |
| facial expression [treat] | -0.003 | 0.125 | -0.024 | 0.981 |
| age | -0.178 | 0.081 | -2.193 | **0.023** |

**Note**: Including an interaction between photoset and facial expression improves model fit ($\chi^{2}$ = 18.216; *p* < 0.001).

| **Table S7.** Coefficients of model describing patterns in looking time to conspecific male photographic stimuli of female rhesus macaques, excluding the main effect of photoset. Brackets indicate the group level. All numerical predictors are Z-standardized, and all categorical variables are effect-coded. Bolded *p* values represent statistical significance at the 0.05 level. | | | | |
| --- | --- | --- | --- | --- |
| **Predictor** | **Coefficient** | **SE** | ***z* value** | ***p*-value** |
| *Male stimuli (n = 292 trials)* |  |  |  |  |
| intercept | 1.270 | 0.579 | 2.192 | **0.028** |
| outgroup [yes] | -0.620 | 0.579 | -1.071 | 0.284 |
| facial expression [treat] | 0.025 | 0.124 | 0.201 | 0.841 |
| age | -0.185 | 0.082 | -2.243 | **0.025** |

**Note**: Including the main effect of photoset does not improve model fit ($\chi^{2}$ = 1.833; *p* = 0.176).

*Model describing patterns in threat sensitivity score*

| **Table S8:** Coefficients of the models describing patterns in threat sensitivity difference score to conspecific photographic stimuli of female rhesus macaques. Brackets indicate the factor level. Bolded *P* values represent statistical significance at the 0.05 level. | | | | |
| --- | --- | --- | --- | --- |
| **Predictor** | **Coefficient** | **SE** | ***z* value** | ***p*-value** |
| *Female stimulus (N = 221 test sessions)* |  |  |  |  |
| intercept | 1.152 | 1.340 | 0.859 | 0.390 |
| photoset [2] | -0.013 | 0.308 | -0.042 | 0.966 |
| outgroup [yes] | -1.374 | 1.324 | -1.038 | 0.299 |
| age | 0.258 | 0.152 | 1.693 | 0.091 |
|  |  |  |  |  |
| *Male stimulus (N = 146 test sessions)* |  |  |  |  |
| intercept | 0.520 | 1.973 | 0.264 | 0.792 |
| photoset [2] | -2.280 | 0.467 | -4.883 | **<0.001** |
| outgroup [yes] | 0.577 | 1.948 | 0.296 | 0.767 |
| age | 0.083 | 0.229 | 0.363 | 0.717 |

| **Table S9.** Coefficients of the model describing patterns in threat sensitivity difference score to conspecific photographic stimuli of female rhesus macaques, excluding the main effect of photoset. Brackets indicate the factor level. Bolded *P* values represent statistical significance at the 0.05 level. | | | | |
| --- | --- | --- | --- | --- |
| **Predictor** | **Coefficient** | **SE** | ***z* value** | ***p*-value** |
| *Male stimuli (n = 146 sessions)* |  |  |  |  |
| intercept | -1.918 | 2.054 | -0.934 | 0.350 |
| outgroup [yes] | 2.092 | 2.067 | 1.012 | 0.311 |
| age | 0.097 | 0.261 | 0.370 | 0.711 |

**Note**: Including photoset improves model fit ($\chi^{2}$ = 21.819; *p* < 0.001).

**C. Reproductive state and socioemotional attention**

*Sample size:*

**Table S10**. Female rhesus macaque subjects at each reproductive state. The 8 nonbreeders that did not skip the birth season correspond to 7 instances of failed breeders who had a baby but the infant died before the test occurred and 1 instance of a late birth (>6 months from test date). The two gestating females that skipped the birth season correspond to stillbirths.

| **Current reproductive state** | **Skipped birth season** | |
| --- | --- | --- |
|  | **No** | **Yes** |
| **Nursing** | 60 | 0 |
| **Gestating** | 70 | 2 |
| **Nonbreeding** | 8 | 59 |

*Pair-wise differences analyses:*

| **Table S11**. Pair-wise differences of looking time to female photographic stimuli across reproductive states. Results are averaged over the levels of facial expression and presence of yearling. Bolded *P* values represent statistical significance at the 0.05 level. | | | |
| --- | --- | --- | --- |
|  | estimate | SE | ***p*-value** |
| nursing - gestating | -0.350 | 0.151 | 0.053 |
| nursing - nonbreeding | -0.577 | 0.149 | **<0.001** |
| gestating - nonbreeding | -0.227 | 0.133 | 0.202 |

| **Table S12**. Pair-wise differences of looking time to a male photographs across reproductive states. Results are averaged over the levels of photoset, facial expression and presence of yearling. | | | |
| --- | --- | --- | --- |
|  | estimate | SE | ***p*-value** |
| nursing - gestating | -0.157 | 0.190 | 0.686 |
| nursing - nonbreeding | -0.365 | 0.185 | 0.118 |
| gestating - nonbreeding | -0.208 | 0.174 | 0.456 |

*Models describing patterns in looking time:*

*Effect-coded factors model:*

| **Table S13**. Coefficients of the model describing relationships between looking time to conspecific male photographic stimuli and experimental variables, age, reproductive state, and presence of a yearling of female rhesus macaques. Models include the random intercept of individual ID. All numerical predictors are Z-standardized, and all categorical variables are effect-coded. Brackets represent the group level, and “X” represents an interaction. Bolded *p* values represent statistical significance at the 0.05 level. | | | |
| --- | --- | --- | --- |
| **Variable** | **estimate** | **SE** | ***p-value*** |
| *Male stimulus (N = 292 trials)* |  |  |  |
| intercept | 0.620 | 0.091 | **<0.001** |
| photoset [1] | 0.116 | 0.073 | 0.112 |
| facial expression [neutral] | 0.043 | 0.060 | 0.477 |
| age | -0.188 | 0.081 | **0.020** |
| reproductive state [nursing] | -0.174 | 0.111 | 0.116 |
| reproductive state [gestating] | -0.017 | 0.104 | 0.872 |
| presence of yearling [no] | 0.048 | 0.076 | 0.525 |
| photoset [1] X facial expression [neutral] | -0.255 | 0.060 | **<0.001** |

| **Table S14**. Coefficients of the main effects only model describing relationships between looking time to conspecific male photographic stimuli and experimental variables, age, reproductive state, and presence of a yearling of female rhesus macaques. Models include the random intercept of individual ID. All numerical predictors are Z-standardized, and all categorical variables are effect-coded. Brackets represent the group level, and “X” represents an interaction. Bolded *p* values represent statistical significance at the 0.05 level. | | | |
| --- | --- | --- | --- |
| **Variable** | **estimate** | **SE** | ***p-value*** |
| *Male stimulus (N = 292 trials)* |  |  |  |
| intercept | 0.622 | 0.178 | **<0.001** |
| photoset [2] | -0.218 | 0.153 | 0.153 |
| facial expression [threat] | -0.014 | 0.124 | 0.908 |
| age | -0.185 | 0.083 | **0.026** |
| reproductive state [gestating] | 0.173 | 0.193 | 0.372 |
| reproductive state [nonbreeder] | 0.397 | 0.189 | **0.036** |
| presence of yearling [yes] | -0.100 | 0.155 | 0.518 |

**Note**: Including an interaction between photoset and facial expression improves model fit ($\chi^{2}$ = 17.529; *p* < 0.001).

**Note**: Including the main effect of photoset does not improve model fit ($\chi^{2}$ = 2.020; *p* = 0.155).

| **Table S15**. Coefficients of the model describing relationships between looking time to conspecific male photographic stimuli and experimental variables, age, reproductive state, and presence of a yearling of female rhesus macaques, excluding the main effect of photoset. Models include the random intercept of individual ID. All numerical predictors are Z-standardized, and all categorical variables are effect-coded. Brackets represent the group level, and “X” represents an interaction. Bolded *p* values represent statistical significance at the 0.05 level. | | | |
| --- | --- | --- | --- |
| **Variable** | **estimate** | **SE** | ***p-value*** |
| *Male stimulus (N = 292 trials)* |  |  |  |
| intercept | 0.510 | 0.160 | **<0.01** |
| facial expression [threat] | 0.016 | 0.123 | 0.896 |
| age | -0.191 | 0.084 | **0.023** |
| reproductive state [gestating] | 0.167 | 0.195 | 0.391 |
| reproductive state [nonbreeder] | 0.354 | 0.188 | 0.059 |
| presence of yearling [yes] | -0.060 | 0.155 | 0.698 |

*Models describing patterns in threat sensitivity score:*

| **Table S16**. Coefficients of the models describing relationships between threat sensitivity score and experimental variables, age, reproductive state, and presence of a yearling. Models include the random intercept of individual ID. All numerical predictors are Z-standardized. Brackets represent the factor level. Bolded *p* values represent statistical significance at the 0.05 level. | | | |
| --- | --- | --- | --- |
| **Predictor** | **coefficient** | **SE** | ***p*-value** |
| *Female stimulus (N = 221 test sessions)* |  |  |  |
| intercept | -0.361 | 0.307 | 0.240 |
| age | 0.268 | 0.157 | 0.088 |
| gestating [gestating] | 0.020 | 0.403 | 0.960 |
| nonbreeding [nonbreeder] | 0.105 | 0.397 | 0.792 |
| presence of yearling [yes] | 0.173 | 0.335 | 0.605 |
|  |  |  |  |
| *Male stimulus (N = 146 test sessions)* |  |  |  |
| intercept | 1.006 | 0.480 | **0.036** |
| photoset [2] | -2.477 | 0.461 | **<0.001** |
| age | 0.039 | 0.230 | 0.866 |
| gestating [gestating] | 0.029 | 0.594 | 0.961 |
| nonbreeding [nonbreeder] | 1.070 | 0.575 | 0.063 |
| presence of yearling [yes] | -0.381 | 0.488 | 0.435 |

| **Table S17**. Coefficients of the model describing relationships between threat sensitivity score towards conspecific male photographic stimuli and experimental variables, age, reproductive state, and presence of a yearling, excluding the main effect of photoset. Models include the random intercept of individual ID. All numerical predictors are Z-standardized. Brackets represent the factor level. Bolded *p* values represent statistical significance at the 0.05 level. | | | |
| --- | --- | --- | --- |
| **Predictor** | **coefficient** | **SE** | ***p*-value** |
| *Male stimulus (N = 146 test sessions)* |  |  |  |
| intercept | -0.136 | 0.472 | 0.773 |
| age | 0.053 | 0.265 | 0.843 |
| gestating [gestating] | -0.018 | 0.651 | 0.978 |
| nonbreeding [nonbreeder] | 0.700 | 0.618 | 0.257 |
| presence of yearling [yes] | 0.067 | 0.531 | 0.900 |

**Note**: Including the main effect of photoset improves model fit ($\chi^{2}$ = 25.992; *p* < 0.0001).

*Reproductive state effects after excluding failed breeders:*

| **Table S18**. Coefficients of the models describing relationships between looking time to conspecific photographic stimuli and experimental variables, age, reproductive state, and presence of a yearling of female rhesus macaques after excluding failed breeders from the analysis. Models include the random intercept of individual ID. All numerical predictors are Z-standardized. Brackets represent the factor level, and “X” represents an interaction. Bolded *p* values represent statistical significance at the 0.05 level. | | | |
| --- | --- | --- | --- |
| **Variable** | **estimate** | **SE** | ***p*-value** |
| *Female face (N = 428 trials)* |  |  |  |
| intercept | 0.368 | 0.124 | **<0.01** |
| facial expression [threat] | -0.175 | 0.089 | **0.049** |
| age | -0.315 | 0.075 | **<0.001** |
| reproductive state [gestating] | 0.391 | 0.151 | **<0.01** |
| reproductive state [nonbreeding] | 0.635 | 0.153 | **<0.001** |
| presence of yearling [yes] | -0.109 | 0.120 | 0.364 |
|  |  |  |  |
| *Male face (n = 278 trials)* |  |  |  |
| intercept | 0.409 | 0.178 | **0.022** |
| photoset [2] | 0.272 | 0.196 | 0.165 |
| facial expression [threat] | 0.389 | 0.160 | **0.015** |
| age | -0.197 | 0.082 | **0.017** |
| reproductive state [gestating] | 0.172 | 0.192 | 0.369 |
| reproductive state [nonbreeding] | 0.418 | 0.193 | **0.031** |
| presence of yearling [yes] | -0.116 | 0.159 | 0.467 |
| photoset [2] X facial expression [threat] | -0.985 | 0.248 | **<0.001** |

*Effect-coded factors model:*

| **Table S19**. Coefficients of the models describing relationships between looking time to male conspecific photographic stimuli and experimental variables, age, reproductive state, and presence of a yearling of female rhesus macaques after excluding failed breeders from the analysis. Models include the random intercept of individual ID. All numerical predictors are Z-standardized, and all categorical variables are effect-coded. Brackets represent the group level, and “X” represents an interaction. Bolded *p* values represent statistical significance at the 0.05 level. | | | |
| --- | --- | --- | --- |
| **Variable** | **estimate** | **SE** | ***p*-value** |
| *Male face (n = 278 trials)* |  |  |  |
| intercept | 0.632 | 0.094 | **<0.001** |
| photoset [1] | 0.110 | 0.075 | 0.143 |
| facial expression [neutral] | 0.052 | 0.062 | 0.403 |
| age | -0.197 | 0.082 | **0.017** |
| reproductive state [nursing] | -0.198 | 0.114 | 0.083 |
| reproductive state [gestating] | -0.024 | 0.106 | 0.818 |
| presence of yearling [no] | 0.058 | 0.079 | 0.467 |
| photoset [1] X facial expression [neutral] | -0.246 | 0.062 | **<0.001** |

**D. Next-year reproduction**

| **Table S20 Likelihood of next-year reproduction based on current reproduction.** Coefficients of the models describing the probability of next-year reproduction as a function of demographic and cognitive variables. “^2^” denotes a quadratic term. Models include the random intercept of individual ID. Brackets represent the factor level. All numerical predictors are Z-standardized. Bolded *p* values represent statistical significance at the 0.05 level | | | |
| --- | --- | --- | --- |
| **Predictor** | **coefficient** | **SE** | ***p*-value** |
| *Female stimulus (N = 199 test sessions)* |  |  |  |
| intercept | 1.382 | 0.288 | **<0.001** |
| age | -0.332 | 0.187 | 0.076 |
| age^2^ | -0.697 | 0.251 | **0.005** |
| skipped current birth season [yes] | 0.417 | 0.509 | 0.413 |

**E. Model diagnostics**

No significant model assumption violations were found through the analyses except for the model testing associations between reproductive state and threat sensitivity score towards a female stimulus. However, we found largely the same conclusions as when modeling associations between sensitivity score towards a male stimulus (i.e., no significant association with reproductive state).

*Evaluation of multicollinearity:*

| **Table S21.** Variance inflation factor for the models describing the associations between reproductive state and looking time to conspecific faces of female rhesus macaques. | | |
| --- | --- | --- |
| **Term** | **VIF** | **95% CI** |
| *Female stimulus (N = 442 trials)* |  |  |
| facial expression | 1.00 | 1.00, 8x46^6^ |
| age | 1.07 | 1.01, 1.31 |
| reproductive state | 1.30 | 1.19, 1.49 |
| presence of yearling | 1.28 | 1.17, 1.47 |
|  |  |  |
| *Male stimulus (N = 292 trials)* |  |  |
| photoset | 1.78 | 1.55, 2.12 |
| facial expression | 1.77 | 1.54, 2.11 |
| age | 1.05 | 1.00, 1.58 |
| reproductive state | 1.27 | 1.15, 1.51 |
| presence of yearling | 1.26 | 1.14, 1.50 |
| photoset X photo expression | 2.50 | 2.12, 3.01 |

As facials expression showed VIF 95% CI > 5, we fitted the same model without this predictor and relative effects sizes and SE remained similar:

| **Table S22.** Coefficients of the model describing looking time to a female face as a function of age and reproductive state after removing the facial expression predictor. The model includes the random intercept of individual ID. All numerical predictors are Z-standardized. Brackets represent the factor level. | | | |
| --- | --- | --- | --- |
| **Variable** | **estimate** | **SE** | ***p*-value** |
| *Female face (N = 442 trials)* |  |  |  |
| intercept | 0.300 | 0.116 | **<0.01** |
| age | -0.306 | 0.072 | **<0.001** |
| reproductive state [gestating] | -0.337 | 0.117 | 0.635 |
| reproductive state [nonbreeding] | -0.562 | 0.151 | **0.025** |
| presence of yearling [yes] | -0.056 | 0.148 | **<0.001** |

| **Table S23.** Variance inflation factor for the models describing the associations between reproductive state and threat sensitivity difference score of female rhesus macaques. | | |
| --- | --- | --- |
| **Term** | **VIF** | **95% CI** |
| *Female stimulus (N = 221 test sessions)* |  |  |
| age | 1.07 | 1.01, 1.59 |
| reproductive state | 1.17 | 1.06, 1.46 |
| presence of yearling | 1.15 | 1.05, 1.45 |
|  |  |  |
| *Male stimulus (N = 146 test sessions)* |  |  |
| photoset | 1.04 | 1.00, 3.66 |
| age | 1.04 | 1.00, 2.86 |
| reproductive state | 1.20 | 1.07, 1.58 |
| presence of yearling | 1.19 | 1.06, 1.57 |

| **Table S24**. Variance inflation factor for the model describing the associations between socioemotional attention to a female photographic stimuli and the probability of next-year reproduction of female rhesus macaques. “^2^” denotes a quadratic term. | | |
| --- | --- | --- |
| **Term** | **VIF** | **95% CI** |
| age | 1.09 | 1.02, 1.51 |
| age^2^ | 1.08 | 1.01, 1.54 |
| skipped prior birth season | 1.07 | 1.01, 1.59 |
| looking time to the neutral face | 1.46 | 1.27, 1.80 |
| threat sensitivity score | 1.40 | 1.22, 1.72 |

| **Table S25**. Variance inflation factor for the model describing the associations between socioemotional attention to a male photographic stimuli and the probability of next-year reproduction of female rhesus macaques. “^2^” denotes a quadratic term. | | |
| --- | --- | --- |
| **Term** | **VIF** | **95% CI** |
| age | 1.18 | 1.06, 1.59 |
| age^2^ | 1.29 | 1.12, 1.68 |
| presence of yearling | 1.09 | 1.01, 1.74 |
| looking time to the neutral face | 1.67 | 1.39, 2.16 |
| threat sensitivity score | 1.62 | 1.35, 2.09 |
